# The secret life of all-or-nothing thinking with exercise: new insights into an overlooked barrier

**DOI:** 10.1186/s12889-025-25780-9

**Published:** 2025-12-19

**Authors:** Michelle L. Segar, John A. Updegraff, Alexis McGhee-Dinvaut, Jennifer M. Taber

**Affiliations:** 1 Institute for Research on Women and Gender, University of Michigan, 204 S. State St, Ann Arbor, 48109 MI USA; 2 Department of Psychological Sciences, Kent State University, 600 Hilltop Dr, Kent, OH 44242 USA

**Keywords:** Physical activity, Self-control, Intentions, Dichotomous thinking, Affect, Role conflict, Decision making, Health behavior

## Abstract

**Background:**

Most people who try to regularly exercise relapse. All-or-nothing thinking with exercise—defined as occurring when an individual is not willing or cannot adhere perfectly to their exercise intentions or plan and chooses to not exercise at all rather than modify the plan—is a potential barrier to sustained exercise that has received virtually no attention within the exercise, public health, and behavioral science literatures. The purpose of this study was to investigate all-or-nothing thinking among individuals who have tried but failed to stick with exercising, with a specific interest in whether and how all-or-nothing thinking may contribute to decisions to *not* exercise when a plan is disrupted.

**Methods:**

Four focus groups were conducted among adults (89% female, 85% white) in two life stages (students, community members) who indicated that they had tried to regularly exercise but had not been able to stick with it (*N* = 27). Data were analyzed using reflexive thematic analysis.

**Results:**

We developed four themes: (1) Rigid idealized exercise criteria lay the groundwork for all-or-nothing thinking; (2) Seeking excuses not to exercise; (3) Exercise is expendable; and (4) Baffled by current inactivity in light of past exercise positivity. Overall, participants appeared to endorse all-or-nothing thinking, and this thinking style seemed to be supported by a set of implicit and explicit cognitive-motivational forces that converged into a greater culturally conditioned mindset that devalued exercising and cultivated rigid self-regulatory practices, guiding participants to opt out of exercising when their plans faced common challenges and competing goals.

**Conclusions:**

Exercise-related all-or-nothing thinking seemed to exist among individuals who have tried but failed to sustain regular exercise and warrants more research. Findings can inform next-stage research and interventions to combat all-or-nothing thinking within public health, behavioral medicine, and community contexts.

**Supplementary Information:**

The online version contains supplementary material available at 10.1186/s12889-025-25780-9.

## Background

Regular physical activity (PA) confers numerous benefits for physical health (e.g. prevents many cancers, improves cardiovascular and metabolic health), mental well-being, and quality of life [1]. Despite this, physical inactivity has increased between 2010 and 2022 worldwide [2], and people have difficulty increasing PA and exercising in ways that they can sustain, even when they participate in theory-based interventions [3].

While research has found that most people report having intentions to exercise (78%), these intentions often fail to translate into engaging in exercise (i.e., the “gap between intentions and behavior”) [4]. Forty-six percent of those who intend to exercise do not successfully follow through [4]. Among those who initiate exercise, the majority relapse within a short time [5, 6]. Compared to people who succeed at regular exercise, those who relapse use fewer strategies to cope with their barriers [5] and have a smaller self-regulatory strategy repertoire , which is associated with less goal attainment [7]. Thus, self-regulatory strategies appear critical for sustainable behavior, including exercise participation [7, 8].

Self-regulation refers to a set of processes that people use to align their behavior with their long-term goals [9, 10] including but not limited to self-control, decision-making, and goal-directed behavior. Self-regulation can involve planning, overcoming temptations, coping with and navigating barriers, managing lapses, and avoiding relapses [11, 12]. Prospective self-regulatory processes—planning the when, where, what and how of exercise—increase the probability of following through on exercise intentions [8, 13]. However, exercise plans often conflict with other life demands, and impromptu decisions at these points of conflict influence whether people engage in exercise [8]. At these decision points, people’s self-regulatory decisions are influenced by the perceived value of that choice within the context of other competing options and desires [14–16]. In addition, experiencing even a single self-regulatory setback makes people more prone to subsequent self-regulatory failure [17]. These setbacks may be due to common barriers such as low self-efficacy, self-regulatory depletion, or lack of time. However, one potential barrier to self-regulation has received virtually no attention within the exercise, public health, and behavioral science literatures: all-or-nothing thinking with exercise.

All-or-nothing thinking has been referred to as dichotomous, black-and-white, and absolutist thinking and is typically considered a formal cognitive distortion that results in rigid processing of information and non-optimal behavioral decision-making [18, 19]. Precise definitions of constructs and their functional role are critical [20] and there is currently no formal definition for exercise-related all-or-nothing thinking in the literature. Therefore, we propose the following definition: "All-or-nothing thinking with exercise occurs when an individual is not willing or cannot adhere perfectly to their exercise intentions or plan and chooses not to exercise at all rather than modify the plan." Within the context of exercise, all-or-nothing thinking may thwart the decision to exercise in dynamic situations when a specific exercise plan or intention becomes unworkable – whether due to an unanticipated barrier such as missing the start of an exercise class or due to feeling too tired or unmotivated to achieve the intended exercise.

There are several reasons to expect that all-or-nothing thinking is a significant impediment to regular exercise. First, there is some empirical evidence on related topics. For example, one quantitative study on “exercise-related cognitive errors” found that greater endorsement of the black-and-white thinking cognitive error (all-or-nothing thinking is a specific type of black-and-white thinking) was associated with lower intentions to exercise [21]. Another qualitative study found strong differences between “low active” and “high active” individuals’ beliefs about what constitutes valid exercise that is worth doing [22]. The low active (but not high active) individuals endorsed beliefs about exercise that reflect absolutist thinking, including rigid standards about what makes exercise worth doing (e.g. exercise must be high-intensity and sweat-producing “to count”). In addition, striving to achieve rigid exercise goals is associated with increased pressure and abandoning exercise routines compared to striving toward goals that are more flexible and open [23]. Second, beyond academic research, the fitness industry frequently discusses all-or-nothing thinking as a top reason that people decide not to exercise [24], suggesting it is an unexplored real-world barrier that is important for researchers to take seriously. Thus, it seems critical to investigate whether people do, in fact, think about exercising as *all* or *nothing*, and if so, how this mental model influences decision and self-regulatory processes.

All-or-nothing thinking may occur both pre- and post-intention. For example, someone in the pre-intentional phase might learn that exercising at least 150 minutes per week at moderate intensity is necessary to achieve health benefits. However, because they believe that they cannot achieve that specific standard, they decide not to exercise at all. In contrast to this pre-intentional phase, post-intentional all-or-nothing thinking would occur after an individual has a plan or intentions to exercise. For example, when this person’s specific exercise plan confronts a challenge that prevents them from exercising exactly as planned, they choose to do "nothing" because they cannot achieve the “all” of their plan. Because many people intend to exercise, yet a significant proportion fails to stick with it long-term [4], this study explored post-intentional all-or-nothing thinking.

Despite indications that rigid, all-or-nothing thinking might undermine exercise self-regulation and decision-making, little is known about this type of thinking as it pertains to exercise. Similar to the maladaptive ways all-or-nothing thinking has been found to function with eating [25], all-or-nothing thinking with exercise could create rigid idealized standards and self-regulatory strategies that are hard to achieve and sustain, especially within the context of other competing daily goals [22]. This is the first in-depth investigation into exercise-related all-or-nothing thinking among people who are not sustaining regular exercise despite having intentions to do so [26]. We used focus groups to investigate whether all-or-nothing thinking was discussed in conversations about exercising among people who have tried to exercise but failed to stick with it, and to explore potentially relevant self-regulatory and decision-making processes, especially when exercise plans and intentions face conflicts and competing goals.

## Methods

### Methodological framework

This cross-sectional qualitative study was grounded in ideas aligned with relativist ontology and non-positivist and contextualist epistemology [27]. As such, we sought to “explore meaning in context” [27] and our research was based on the perspective that knowledge differs across contexts and that there is no single underlying truth. Further, we assumed that people’s exercise experiences are socially constructed and shaped through an interplay of individual perceptions as well as the greater cultural narratives, norms, and pressures [27–29].

### Sample and recruitment

We conducted four focus groups: two with community members recruited from the city of a Midwestern university and two with undergraduate students recruited from that university. We chose people in these two life stages because it allowed us to explore whether adults living in distinct life stages had similar or different experiences with the questions we were investigating. Recruitment took place through flyers, electronic screens on that university’s campus, local newspaper advertisements, and through the Department of Psychological Sciences’ subject pool.

Because almost half of people who intend to exercise do not successfully follow through [4], we sought to investigate all-or-nothing thinking among these “unsuccessful intenders.” Our advertisements recruited individuals who resonated with having “tried to regularly exercise” and “just couldn’t stick with it.” Advertisements directed interested participants to an online survey and survey responses were reviewed for eligibility criteria and availability to attend one of the pre-scheduled groups. Eligibility criteria were: at least 18 years of age, fluent in English, reported engaging in < 30 min of exercise in the past week and that this amount was “about the same as usual,” responded that they had not started a new exercise routine in the past week, and indicated that the statement, “I am someone who has often tried to exercise regularly, but then finds that I can’t stick to my exercise plans” was “very” or “somewhat true for me.” Of note, we did not define exercise either in the recruitment materials or the focus groups because it was important for us to ascertain participants’ own definitions of exercise as it related to our research aims. Twenty-seven people (11 students, 16 community members) participated across four focus groups (ranging from 3 to 9 participants in each). The sociodemographic characteristics of participants are shown in Table 1.

**Table 1:** Participant sociodemographic factors stratified by student versus community groups

Sociodemographic factors	Community participants (*n* = 16)		Student participants (*n* = 11)		Total (*n* = 27)	
	n	%	n	%	n	%
Gender						
Male	1	6.3	1	9.1	2	7.4
Female	15	93.8	9	81.8	24	88.9
Nonbinary	0	0	1	9.1	1	3.7
Education						
High school or less	0	0	0	0	0	0
Post high school training other than college (vocational or technical)	0	0	2	18.2	2	7.4
Some college	4	25.0	9	81.8	13	48.1
College graduate	8	50.0	0	0	8	29.6
Postgraduate	4	25.0	0	0	4	14.8
Ethnicity						
White	15	93.8	8	72.7	23	85.2
Black or African American	0	0	2	18.2	2	7.4
Asian	0	0	1	9.1	1	3.7
Rather not report	1	6.3	0	0	1	3.7
	M (SD)	Range	M (SD)	Range	M (SD)	Range
Age	55.4 (17.1)	26 to 79	19.8 (1.1)	19 to 22	40.9 (22.1)	19 to 79

Our sample size was determined by financial resources and participant interest and eligibility. We believe this sample has sufficient “information power” because of the specificity of our study aims and sample, our use of established theory in question design, and because of the rich, in-depth participant conversations and our rigorous analytic process [30], described below. We had not previously met any of the participants in this sample.

### Data collection and process

Our primary goal was to explore whether participants who had tried to exercise (e.g., had intentions) but failed to stick with it discussed exercising in ways that reflected the use of all-or-nothing thinking, in addition to understanding their self-regulatory and decision-making processes.

We conducted a qualitative inquiry using focus groups because qualitative research is inherently flexible and permits exploring new issues that arise during the data collection process [31–33], making it ideal for investigating a topic about which little is known, such as all-or-nothing thinking with exercise. Our semi-structured focus group guide allowed us to engage in unplanned conversations that emerged organically during the group conversations. Prior to the focus groups, we pilot tested and refined the questions by soliciting feedback on meaning and clarity from non-academics and in a lab meeting, as well as through multiple discussions among the full research team. During each focus group, when participants didn’t understand a question, we rephrased it [33]. Between groups, we further evaluated and modified questions that did not elicit meaningful responses, allowing us to ask refined questions to better assess the phenomenon of inquiry [33, 34]. Some ways in which the questions changed between groups included removing questions that didn’t resonate with participants, rewording some questions, and changing the order of questions (e.g., asking about exercise barriers near the beginning of the first focus group versus asking about plan disruptors near the end of the fourth focus group). As an example of a reworded question, to understand what participants thought counted as sufficient exercise, we initially asked this directly: “How do you think you should exercise to make it worth doing?” In the fourth group, we sought to elicit perceptions of this in a more indirect way: “Please take a minute to remember a time when you’ve started to try to exercise. (PAUSE) At the times you’ve tried to start exercising again, what do you do for exercise?” The final guide that we used with the fourth focus group is available in Supplemental Materials. 

Our focus group questions were informed by general ideas from self-regulation, motivation, goal striving, and decision-making research [9, 16, 35] and were written to elicit participants’ beliefs about, criteria and plans for, and barriers, motivators, and approaches to and strategies for exercising more generally. Specific questions assessed participants’ immediate single-word association with exercising and their primary motivation for starting to exercise (i.e., “In general, what has been your primary reason for wanting to exercise and why?”). Questions were included to ascertain participants’ perceptions about “what counts” as exercise. Questions also targeted participants’ behavioral choices in response to barriers (e.g., “At these moments when exercise becomes challenging, what types of things make it hard to stick with it?”) and disruptors of specific plans (e.g., “When you think about times when you’ve had a specific plan to exercise, what types of things tend to get in the way of or disrupt your plan?”). In addition to understanding *what* derailed participants’ exercise plans, questions probed participants’ internal narratives and self-regulatory processes at points of decision-making about an intended exercise session, especially when faced with competing goals or other conflicts. (“Let’s say that you’re about to start your planned exercise or about to leave to go do it. Imagine you get an unexpected text that requires you to do something right then that takes 20 minutes. What do you typically do about that planned exercise session?”). We also asked questions to understand participants’ processes and motives related to starting to try to regularly exercise and stopping (“We’re talking about starting and stopping exercise. Since exercise can be challenging to stick with, why do you think you keep trying to exercise?”).

### Data analysis

We used a reflexive thematic analysis process to explore the potential existence of exercise-related all-or-nothing thinking and to identify meaningful patterns in people’s self-regulatory and decision-making processes. Reflexive thematic analysis is theoretically flexible, permitting investigators to analyze patterns of shared meaning while concurrently maintaining awareness of their respective assumptions and experiences [27]. While the data analysis took into consideration participants’ explicit comments (e.g., semantic analysis), we were especially interested in the implicit meanings and assumptions underlying participants’ comments (e.g., latent analysis). Thus, we played an active role in determining the meaning of the data by analyzing participants’ comments and expressed attitudes and beliefs within the broader cultural context (e.g., history of exercise recommendations in the U.S.). We believe that the themes we generated from the data are not contingent or dependent on the particular questions asked or the order questions were asked in, as questions were designed to be both broad and specific in eliciting participants’ understandings of how they related to exercising and responded to barriers. Our analysis also included both inductive and deductive components [27]. Deductively, we explored whether participants’ narratives about their exercise experiences included statements reflecting all-or-nothing thinking. Inductively, we sought to understand the ways in which participants discussed their decisions and self-regulatory processes as they related to following through with exercise intentions or plans, especially when confronting challenges.

Because the focus groups consisted of participants in two life stages (university students with a mean age of 20 years and community participants with a mean age of 55 years), we initially assessed the data separately by life stage to permit identifying content that might be distinct and/or similar between these two groups. We began our analysis with the student focus groups. We independently reviewed the transcripts and generated a list of initial codes accompanied by the relevant data each code was based on, which we then exchanged, reviewed, and discussed. We then integrated our respective codes to assess how they fit together. Then, we repeated the same analytic method with the data from the community focus groups, after which we organized and evaluated the codes across the student and community groups to explore potential themes from the full dataset. Throughout this process, we went back and forth numerous times between the code sets, tentative themes, and the raw data, comparing and contrasting findings across the student and community groups. This iterative process of thematic meaning-making and refinement occurred over multiple months through speaking, writing, visual mapping, and the manuscript peer-review process until we settled on a final set of themes that best fit the data. We organized these themes into a framework that meaningfully reflected the findings as they related to our research questions [27].

We (the first and last authors) fostered rigor and trustworthiness through participating in critical dialogue (i.e., "critical friends") throughout the analytic process [33]; asking the third author, who was present for each focus group and took extensive notes, for her review and input; and discussing the data with the second author. We considered our data from multiple alternative perspectives and had additional in-depth conversations about these data with numerous other academics studying related topics. Through the final writing of this paper, we reflexively challenged each other, comparing and contrasting our individual interpretations, always returning to the transcripts or original recordings of the focus groups to inform our interpretation of the data.

### Reflexivity

Both authors conducting the analysis (Authors 1 and 4) are psychologists, trained in personality and social psychology, respectively, and who aim to be regularly physically active. In addition to being a behavioral scientist, Author 1 has been a health-coach practitioner for many years, working with clients who intend to exercise but have struggled numerous times to sustain an exercise routine. Our distinct professional perspectives and personal experiences exercising contributed to investigator triangulation [27].

## Results

We developed four themes that related to all-or-nothing thinking: (1) Rigid idealized exercise criteria lay the groundwork for all-or-nothing thinking; (2) Seeking excuses not to exercise; (3) Exercise is expendable; and (4) Baffled by current inactivity in light of past exercise positivity. Our findings are organized around these themes and the greater framework integrating them. Participants’ names were changed to ensure their anonymity.

### Theme 1: Rigid idealized exercise criteria lay the groundwork for all-or-nothing thinking

A requirement for all-or-nothing thinking is having an “all.” For most participants, their “all” constituted rigid idealized criteria for exercising. While the standards were not the same for everyone, most participants had some personal definition and criteria for doing exercise “right.” For some, their criteria reflected an idealized duration, such as exercising “for 45 or 60 minutes.” For others, exercising in the right way meant exercising at the gym or exercising for the amount of time that had been planned. Some participants noted that if the timing of their exercise session was thrown off (e.g., they had 20 min less to exercise than planned), spending less time exercising would not be valuable or worth doing:

“If I do something for under 15 minutes, I feel like I didn’t even exercise. Even if it had been dead out sprints, it just doesn’t factor into my head like I did anything.” (Community, Nina).

One student said that while she might celebrate a friend for exercising less than planned, she wouldn’t do it herself:

“…if somebody told me that they went to the gym for 15 minutes, I’d be like, “Rock on. That is good…but I just personally wouldn’t go at all.” (Kayte).

For most participants, if they couldn’t do their exercise exactly as planned, they generally chose to do nothing suggesting they employed all-or-nothing thinking. The following quotes suggest that idealized exercise criteria may create the platform for using all-or-nothing thinking in exercise-related decision-making:

“I have to go for the time I thought I was going to go for or nothing.” (Student, Carli).

“…if I’m being honest, I would like to say that I would do it for less of a time, but it’s either all or nothing.” (Community, Nancy).

Yet, idealized criteria were not only about the duration of exercising. Comments indicated that participants believed that exercise had to be physically effortful—done at high-intensity—to be worth doing:

“If you’re going to work out, just make sure your heart rate gets up. That’s what you want to do is get your heart rate up and keep it up for a consistent amount of time…that’s the whole goal of exercising.” (Student, Elizabeth).

Indeed, even though admitting that this high standard was unachievable for her, one community participant believed that training for marathons was the “right” way to exercise:

“I am imagining my friend who’s training for the Boston Marathon doing it for the third time…she just runs all the time. And for me, that seems like that’s the right way to do it. And I’m not in (sic) that at all. Not attainable or not sustainable sort of thing [for me].” (Community, Kali).

In addition, because their rigid idealized exercise criteria tended to require high-intensity exertion, some participants did not believe that walking “counted” as valuable exercise:

“I feel like it’s not really, like, exercise unless, like, you’re sweating and your heart’s in like [mimics heavy breathing], like out of breath because I walk a lot between classes…I don’t necessarily see that as exercise…It’s just something that I have to do, not really exercise.” (Student, Tresa).

Beyond intensity level and duration of session, participants also talked about the need to exercise on the same days every week, without variation:

“I think it probably needs to be something consistent…Not just like this week I’m going to do Monday and next week I’ll do Thursday afternoon and then the next week I may do Monday, Tuesday, Wednesday. I think it needs to be more consistent.” (Community, Jade).

“Most of the time I’ll stop because if I don’t hit the four to five days, I’ll stop and feel like a failure, then never do it again.” (Student, Kayte).

These data suggested that having rigid idealized criteria frequently resulted in choosing not to exercise.

### Theme 2: Seeking excuses to not exercise

Our second theme revolved around participants, especially in the community groups, noting that they proactively sought reasons to avoid exercising. This active desire to avoid exercising is unsurprising given that many participants experienced high levels of negativity when striving to achieve their often idealized, effortful criteria as the explicit aim for exercising:

“I don’t think it feels good to do. It feels good after. But in the moment I’m like, ‘I hate this.’” (Community, Amy).

“I’m going with it’s hard. It’s hard to breathe. It’s hard. Everything hurts. It’s hard.” (Community, unidentifiable speaker).

The ease with which participants sought excuses not to exercise is exemplified by the two comments below:

“It doesn’t take much of a bump in the road…a piece of sand will get me off just enough to throw me off the momentum of that direction.” (Community, David).

“I can have such determination and follow-through in areas of my life like professional or hobbies that are not based around sweating, but I can talk myself out of going outside [to exercise] any day…” (Community, Nina).

While students generally spoke less negatively about exercising than the community participants, their comments also suggested that some sought excuses to not exercise:

*“*Oh, I didn’t have time today, maybe let’s do it tomorrow. Something comes up tomorrow, oh, okay, let me do it the next day…I just keep pushing it and saying, I will, I will, I will, and I never do.” (Student, Rebecca).

Most student and community participants also perceived exercise as a chore and as something they “should” do. Many participants, especially in the community groups, described body-shaping and weight-related motives for exercising that seemed to exert internal pressure. While they discussed wanting to achieve idealized appearance and weight-related goals through exercise, their related comments were filled with “should” language and pessimism. Participants lamented that they had “let themselves go,” mentioning disappointments such as their continued failure to fit back into their jeans. They also noted feeling pressured by past rebukes about exercise they had received from their doctor, and for some, their own (unrealistic) expectations of weight loss:

“…usually I’m motivated by, I want my figure, I want to lose weight, I want my heart rate better, I want my doctor not to yell at me, blah, blah, blah. And I end up investing, whether it’s money, time, all this willingness, and it’s just too great and I expect too much of a return too soon.” (Community, Joan).

“Exercise, even though it’s enjoyable after you’ve done it, it feels more like a chore… something that you’re forced to do because your doctor said, ‘Do this or you’re going to be taking medication.’” (Community, Nancy).

Against this backdrop of body-shaping disappointments, feeling pressured by clinicians’ warnings, and no external accountability, community participants sought to “rationale [sic] it [exercise] out of existence” (Sharon, Community).

Although for different reasons, students also generally discussed exercising as a “should.” Some mentioned that exercising felt like “school”; something you need to do but don’t want to. Their comments suggested that feeling pressured to exercise derived from having learned about exercise through physical education classes, team sports, and Michelle Obama’s public health initiative, all of which exhorted them to achieve specific exercise and fitness criteria. Yet, despite these earlier controlling exercise contexts, students’ current narratives around exercising to get fit or into shape seemed to be more autonomous and optimistic than community participants’ narratives. In addition, compared to the community participants, students generally had more holistic perspectives about how exercise would help them feel better, emphasizing benefits to their mental health and sense of well-being. Thus, students did not seek excuses to avoid exercising to the same extent as community participants. Their decisions to abandon their exercise plans were less emotionally fraught and more related to the immediate logistical challenge facing their exercise plans, such as needing to study.

### Theme 3: Exercise is expendable

With limited time as the currency for task completion, when their rigid exercise plans bumped up against competing goals—such as school assignments or preparing dinner—most student and community participants said that they typically chose to forgo exercise. In contrast to exercise, participants said that not fulfilling their other daily plans and responsibilities would result in tangible negative consequences. The expendability of exercise was operationalized with a straightforward calculus:

“I think it’s [exercise] expendable in my mind, because if I plan to do laundry on Saturday and it doesn’t get done, I know I have to do it Sunday…You know you have to do laundry or you have to go to the grocery store. You don’t have to exercise.” (Community, Jade).

Participants noted how easily exercising could get pushed out by more important priorities:

“…when…your routine ends up getting crowded and crowded with the things that have to be done or should be done, this [exercising] is an easy thing to push to the side.” (Community, Mary).

“It [exercise] goes first.” (Community, Joan).

“And my sleep schedule is already bonkers because of how much stuff I have to do, there’s just no time for me to miss assignments or to add on this extra stuff at the end of the day, like exercising.” (Student, Rebecca).

The expendability of exercise manifested through inflexible or nonexistent coping strategies when participants’ rigid exercise plans faced an unexpected conflict. Many participants described how when facing competing goals, pivoting and substituting their intended exercise with another physical activity wouldn’t even cross their minds. Others said that even if they had a viable fallback option, they wouldn’t use it. One community participant stated that when she had planned to walk with a friend [the *all*] and the friend canceled at the last minute, she chose to do *nothing* even though she had blocked off that time specifically for walking and had a treadmill in her house:

“I walk with a friend once a week, and if she cancels on me, I have the time blocked. There’s no reason why I can’t just go out and do a walk by myself, but I just won’t go, and I won’t replace it with the treadmill that’s literally in my home.” (Community, Holly).

When a conflict arose to participants’ expendable exercise plans, doing *nothing* was most often the go-to choice. However, there were some exceptions to this all-or-nothing rule: a few participants noted that they would modify their intended activity when it faced a conflict. One student said that if a conflict arose to his planned gym visit, he would likely push back the start time until later that night when he could complete the full duration. Another student showcased her ability to flexibly pivot when her specific exercise plan was derailed:

“We were supposed to go to the gym…so [when that wasn’t feasible]…I did something at home with the yoga mat and weights, it wasn’t much, but it was still something.” (Student, Jenny).

As the above examples suggest, more flexible and dynamic coping strategies, by necessity, were accompanied by more inclusive criteria for "worthy" exercise. Some, but not most, of the participants were able to think beyond the *all* that they had planned to do to identify and pursue the *something* that they could fit in. The comments across the focus groups suggested that exercise plans based on rigid idealized criteria compete with—and most often lose to—other daily goals and priorities.

Interestingly, in contrast to most participants’ reported inflexibility coping with exercise conflicts, some participants across both student and community groups noted that *in other life* areas they are flexible and do pivot their plans when conflicts arise (e.g., meal preparation, social plans). One student said that when it comes to exercising, she doesn’t roll with the punches when it gets interrupted, whereas she is flexible in other life areas:

“I usually go with the flow in my life…But if something doesn’t go my way, I’ll just think in the moment and take in the circumstance and see what the backup plan is…But with the gym it’s like I have to be in the optimal situation to go, otherwise I’m probably not going to go.” (Salafa).

Thus, while inflexible criteria and all-or-nothing thinking seemed to dominate exercise decision-making, it did not extend to other life priorities because those were more highly valued, not considered expendable, and were able to be modified when needed.

### Theme 4: Baffled by current inactivity in light of past exercise positivity

Despite having negative affective experiences while exercising as described in Theme 2, participants also mentioned having had past positive affective experiences with exercise. While some discussed feeling good only *after* exercise was over, such as having more energy and feeling more comfortable in their body, others noted this positivity occurred *while* they were exercising, such as feeling proud, having less anxiety, clearing one’s mind, and having fun with friends. However, across students and community members, most of these positive experiences tended to be recollections of a distant past when participants had regularly exercised. Students’ positive memories often focused on being active with others. For example, one student noted that during high school, exercising was “under the guise of hanging out with your friends” (Student, Jenny) while another emphasized how “fun” being active was with others:

“Maybe when you’re with friends or doing sports or just hanging out. Maybe when you act like a kid, when you’re like, ‘Oh, let’s play tag,’ and everyone starts running around and having fun, you don’t think about it.” (Student, Elizabeth).

Some community members also recalled having had past positive experiences exercising:

“I just love the fitness circuit at the university…a long time ago…I go [went] because that’s my relaxation time… you could just focus and you go from one thing to the next thing…you don’t even have to think about it.” (Community, Ava).

Others recalled how much better they felt when they had regularly exercised:

“I know from experience when I was in a regular exercise routine, I actually had better energy…I slept better…So intellectually I know that it would actually be an improvement to my probably efficiency.” (Community, Holly).

Given these past positive experiences with exercise, some participants were baffled about why they couldn’t get themselves to be regularly active now. One community participant recalled loving the camaraderie she experienced with her former colleagues at the gym, but wondered why she could no longer get herself to exercise after retiring:

“I don’t understand why [I don’t exercise.]…I just had my 70th birthday and I’m in terrific health…But if I don’t start exercising regularly, I’m going to throw [that] away…I’m an educated woman…Why can’t I even make a dent in it?” (Community, Ava).

One student reminisced about how being active in high school was fun:

“Sometimes I feel like reminiscing on sports or fun activities that you did…I enjoyed doing that…Why don’t I do it again?’’ (Elizabeth).

Participants’ comments suggested that they were not consciously aware of the possibility that past cultural conditioning kept them trying to achieve the same rigid idealized exercise criteria that they had succeeded with in the past – even though their life circumstances, activity preferences, and/or body abilities may have changed. While participants discussed having an easier time being regularly active when they had fewer responsibilities – such as before they had children, before they were attending college and working at the same time, or when they participated in structured exercise through the military or cardiac rehab – they didn’t seem to recognize that those past contexts and circumstances may have played a critical support for regularly achieving their idealized exercise criteria. Instead of adapting their exercise standards to better align with their current life roles, schedules, physical abilities, and movement preferences, they blamed themselves for not meeting the former activities and standards that they had once regularly achieved:

“When I did have a regular exercise routine, because I was young and single, I was at the gym five days a week for 60 to 90 minutes each day. And that felt like the right way to do it. I was doing cardio, I was doing weights, I was doing everything. And anything less than that seems like why bother, because I can’t do it the way it should be done. So I know that that’s warped, but yeah.” (Community, Tanda).

“I hadn’t worked out like that since I was in the military…but I can’t seem to get my butt in gear for doing strength training. And I like strength training, but I don’t know what my barrier is for that.” (Community, Rebecca).

In their upward comparisons to their past exercise, participants’ current exercise always came up short. They tended to attribute the inevitability of their exercise plan derailment to a lack of motivation or blamed it on life just getting in the way, instead of considering the possibility that the exercise standards they kept trying to achieve might be overly ambitious, undesirable, or unsustainable within their current life context. Regardless of the reason, when they felt unable and/or unmotivated to achieve these past higher standards, they often chose to do nothing rather than something else.

### Exercise-related all-or-nothing mindset framework

**Fig. 1:**
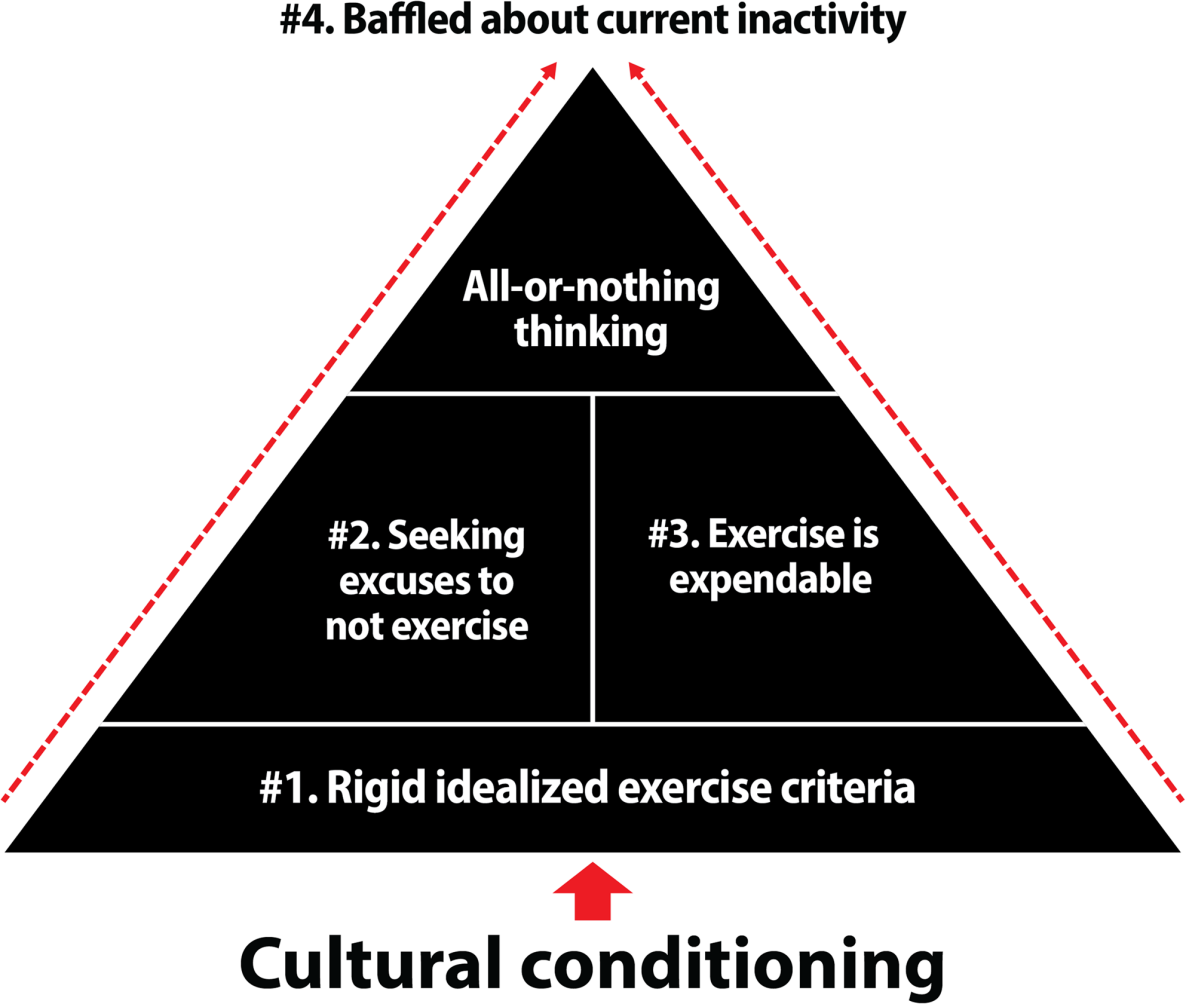
Exercise-related all-or-nothing mindset framework. *Note. This figure specifically reflects the findings from the community participants

We next integrated the four themes from the focus groups into a conceptual framework that reflected how they are interrelated. Figure 1 presents this initial framework; supporting research for this framework appears in the Discussion.

By definition, rigid idealized exercise criteria create the foundation of all-or-nothing thinking because they constitute the “all.” However, these findings suggested that, in addition to rigid idealized exercise criteria being the foundation for all-or-nothing thinking, they also appeared to form the basis for Themes #2 and #3. Indeed, data suggested that participants sought excuses to not exercise because they wanted to avoid the negative experiences and "should"-based pressures that they associated with trying to achieve their rigid idealized criteria (Theme #2). In addition, rigid idealized criteria, by definition, cannot be modified. Thus, because participants didn’t perceive modifying their idealized exercise plan as an acceptable option, they deemed exercising expendable and easy to sacrifice when facing competing priorities that they more highly valued (Theme #3).

Thus, data suggested that exercise-related all-or-nothing thinking constituted more than just a binary thought process. Instead, it appeared to also be supported by a set of culturally informed cognitive-motivational forces (Themes #1, #2, and #3) that collectively fostered an *all-or-nothing mindset*; a mental filter which fundamentally devalued the choice to exercise and also left many participants *baffled by their lack of regular exercise* (Theme #4).

## Discussion

Successfully sustaining exercise requires regularly making in-the-moment decisions that favor being physically active. To do this, people need to think about exercising in ways that can facilitate successfully navigating the barriers that commonly arise to their exercise plans. This research provided support for post-intentional all-or-nothing thinking as a potential impediment to adaptive decision-making and flexible self-regulation. Findings suggested that rigid idealized exercise criteria created the foundation of all-or-nothing thinking and contributed to a greater mindset that devalued exercising.

### Rigid idealized exercise criteria create the foundation of all-or-nothing thinking

Participants generally had idealized criteria for exercising that were rigid and unmalleable, constituting the *all* within exercise-related all-or-nothing thinking. This type of binary thinking is typically considered a formal cognitive distortion [18] or cognitive error [21]. We propose, however, that within the context of exercising, all-or-nothing thinking reflects sound reasoning: People’s decision to do *nothing* when they cannot achieve the *all* reflects a rational choice based on standards for exercising that they have learned within society [36].

 As a society, people have learned there are specific exercise guidelines to follow and intensity and duration criteria to meet (e.g. “exercise at high intensity for at least 60 minutes per week”) [37, 38]. This “threshold messaging” has dominated exercise communications and education for decades [39]. In addition, this cultural conditioning has come from numerous trusted, and thus persuasive, sources [40], including the media, researchers, formal federal guidelines, social media influencers, and clinicians. These messages have been so pervasive that people may have internalized these standards as formal rules that must be followed to “correctly” exercise [41]. Therefore, when they are unable to meet the *all* that they have internalized as necessary for worthy exercise, they might believe that doing anything less would not be valuable or worth doing [37, 41]. In fact, when participants’ plans faced challenges, they rarely chose to modify their plan to do *something* instead of *nothing*.

Recently, updated PA guidelines have acknowledged mounting evidence that all movement is beneficial and that some movement is almost always better than none [1]. However, if people who have been unsuccessful sticking with exercise have internalized rigid criteria as their standard for exercising, it could be challenging to change beliefs about and value the more inclusive alternative of “something is better than nothing,” especially among novice exercisers [42, 43]. More broadly, these data suggest that the reason participants did not close the gap between their intentions and behavior is because their idealized intentions led them to devalue more feasible behavioral alternatives when facing common barriers, which fostered exercise-related all-or-nothing thinking and the associated rigid self-regulatory processes [44, 45]. Based on these findings, it is also possible that our field’s focus on trying to “close” the intention-behavior gap [4] is one reason there has been very little research on all-or-nothing thinking with exercise [26].

All-or-nothing thinking is a critical issue for people who are trying to become regular exercisers because within the binary, the only option when you can’t do it *all* is *nothing*. Thus, when a full exercise plan cannot be fulfilled—regardless of the reason—believing in the need to achieve *rigid idealized exercise criteria* (Theme #1) may devalue other feasible exercise options [42]. In essence, all-or-nothing thinking may thwart people choosing to move whenever situations dynamically occur (e.g., low motivation, competing goals) that prevent them from achieving their *all*. In fact, this proposition is supported by studies showing that more rigid definitions of worthy exercise are associated with less exercise participation compared to more inclusive definitions [46, 47]. All-or-nothing thinking may inhibit the type of flexible self-regulation that mounting research suggests underlies adaptive decision-making for behavioral maintenance within exercise [42, 48], eating [49], and chronic disease management [50].

While our findings are preliminary, other relevant literatures show the maladaptive consequences of all-or-nothing thinking. Within the eating and weight management literature, rigid, dichotomous thinking is a well-documented barrier to adaptive eating decision-making and self-regulation and predicts abandoning weight-loss efforts and weight regain [25, 51]. Dichotomous (all-or-nothing) thinking is theorized to thwart healthy eating decisions and weight management through a variety of mechanisms including contributing to “rigid restraint”—setting strict idealized rules about what and how much to eat, regardless of personal preferences and varying needs related to daily eating. Because it reflects a rigid form of self-regulation, rigid restraint is challenging to sustain [52]. In fact, emerging research, including the new dominant thinking within the self-control literature, indicates that rigid self-regulatory beliefs and processes result in worse outcomes than more flexible ones [44, 53, 54].

Among our participants, rigid idealized exercise criteria seemed to do more than just create the standard for choosing “no go” at the go/no-go decision point. As depicted in Fig. 1, our analysis suggested that *having rigid criteria for exercising* (Theme #1) *also created the basis for other potent cognitive and motivational forces* (Themes #2, #3) *that conjoined*,* below the surface*,* to create an exercise-related all-or-nothing mindset that devalued exercising and resulted in decisions to not exercise*– despite having had *positive experiences exercising in the past* (Theme #4).

### An exercise-related all-or-nothing mindset fosters the devaluation of exercising

Participants, especially in the community focus groups, discussed exercise’s value as a health-promoting, weight-reducing, and gym-attending “should” that they needed external accountability to push themselves to do, but in fact, *sought to avoid* (Theme #2). People have been socialized to believe that to achieve their weight- and health-related goals they must meet *specific criteria when exercising* (Theme #1) [38]. Thus, within the context of these social norms and cultural conditioning, the perceived utility of exercising *less* than these rigid gold standards would be low and could contribute to all-or-nothing thinking [39]. When participants’ exercise intentions faced competing goals and they couldn’t do the *all* they believed was required, they most often chose to sacrifice exercise entirely and do *nothing*. This type of rigid decision-making and self-regulation is concerning given the mounting research showing that flexibility supports sustainable behavior [48, 53, 54] and people who flexibly substitute one exercise plan with another participate more than people who do not make these activity swaps [55].

It is important to consider the perceived expendability of exercising within the context of identity. This construct refers to identifying as “as an exerciser” and perceiving exercising as fostering identity-expressing values and goals, which boosts the personal relevance of exercise and is believed to support behavioral maintenance [56, 57]. Accumulating neuroscientific evidence suggests that people are more likely to follow through with behavioral goals that feel self-relevant in this way because these goals have greater subjective value than identity-irrelevant behaviors [58]. Yet, these data suggested that most students and community participants perceived exercising more as a “should” instead of a highly self-relevant, values-supporting activity [22]. This may be because many strove to achieve effortful, *rigid idealized criteria* (Theme #1) to conform to culturally prescribed health- and body-related norms [22, 59], aspirations which are often associated with psychological costs including internalized weight stigma, body-shame, and compromised mental health [60, 61].

In fact, participants frequently mentioned different types of costs associated with striving to achieve *rigid idealized criteria* (Theme #1). For example, student and community participants perceived exercising as taking too much time and effort away from more valuable priorities and tasks, deeming it *expendable* (Theme #3). This aligns with research showing that people who do not regularly exercise value non-exercise tasks more highly than those who are regular exercisers [11] and that conflicting goals impede following through with intended exercise [62]. While students seemed to value exercising more than community participants, students still described exercise as a low priority [63]. Regardless of life phase, participants overwhelmingly believed that *exercise was expendable* (Theme #3) compared to other daily tasks. This is likely why, when competing needs arose, participants were more willing to be flexible and modify their plans to accomplish tasks in *other* life areas (e.g., such as with school, work, and parenting) but used rigid thinking and self-regulatory processes with exercise.

Exercise was also discussed as having experiential costs derived from negative, effortful experiences, especially among community participants who actively *sought excuses to avoid exercising* (Theme #2) which they “dreaded” and “hated” [14]. Participants’ narratives suggested that this constellation of internal and external costs may have overwhelmed any value that exercise had, which appeared to guide them – consciously and/or non-consciously – towards choosing *nothing* when they had to decide between their all or nothing.

Importantly, participants were *baffled by their lack of current exercise in light of the positive exercise experiences* that many reported having had in previous life stages (Theme #4). This is unsurprising because people are often not aware of how their cultural conditioning, experiences, preferences, beliefs, and values influence their daily decisions [64]. Moreover, mindsets are culturally conditioned, deeply imbedded beliefs, assumptions, and attitudes; mental filters that bias value calculations and influence decisions, often outside of conscious awareness [47, 65, 66]. An exercise-related all-or-nothing mindset may detrimentally influence exercise participation because it guides people to implicitly devalue exercising and hides the specific cognitive-motivational forces that underlie their decisions to do nothing [14, 47, 64–67]. Thus, having an exercise-related all-or-nothing mindset might have a secret life that functions below conscious awareness thwarting decisions to exercise [14, 64, 66].

There is a theory that can help with understanding the gestalt of these findings. The Situated Expectancy-Value Theory (SEVT) [68, 69] is one of the most widely used theories in education science and considered one of the “Big Theories of Motivation,” but has rarely been applied to exercise participation. However, the SEVT is useful in interpreting our findings. Although the SEVT is a comprehensive model with many components, it is valuable for exercise decision-making and behavior because it contends that the value of a choice is influenced by the cultural milieu and accompanying social norms and pressures, as well as the features of the immediate situation [16]. Thus, the SEVT offers a lens for understanding the ways in which people’s cultural conditioning about exercise influences their beliefs, perceptions, experiences, motivation, justifications, and decisions [16, 68]. In addition, similar to newer dual-process exercise theories [14, 70, 71], the SEVT assumes that decisions are situated within dynamic in-the-moment contexts (i.e., social, emotional, physical) and are enacted through both reflective and automatic processes [65, 69].

Through its “subjective task value” construct, the SEVT contends that the aggregate value of choosing an activity over its alternatives is created by calculating (implicitly and explicitly) the pros of a behavioral choice minus the cons. Thus, the subjective task value of exercising would be the “net” value of exercising in any given moment, calculated by the sum of valued aspects minus the perceived costs associated with exercising [16]. The subjective task value construct offers insights into how the explicit and implicit costs of exercising could have overwhelmed participants’ rational intentions to exercise, making the decision to do *nothing* a prudent choice and desirable exit strategy. Furthermore, because value calculations often occur below conscious awareness [67, 69], it makes sense that participants could be *baffled by their inactivity* (Theme #4) despite the explicit value they believed it had for them.

To increase the perceived value of exercising and reduce all-or-nothing thinking, these data suggest a need to fundamentally reorient people to a new “what,” “why,” and “how” of exercising. This might be achieved through guiding people to: (1) strive toward more realistic exercise standards and positive experiences that reflect intrinsically motivating preferences instead of prescriptive gold standards [14, 35, 38]; (2) perceive exercise as a way to immediately serve identity-supporting goals, daily priorities, and quality of life instead of socially prescribed body-related norms and pressures [29, 56, 58], and; (3) use flexible instead of rigid self-regulatory and decision processes when exercise intentions and plans face conflicts [22, 42, 54, 55]. In addition, an exercise-related all-or-nothing mindset might influence people’s exercise decisions to avoid exercising outside of conscious awareness. Thus, to better support sustainable exercise participation, interventions should raise participants’ awareness about how cultural conditioning can negatively impact their exercise-related beliefs, valuation processes, and decisions and offer more positive alternatives [22, 29, 41, 72].

### Other considerations and future directions

This research offers new insights and preliminary theorizing on all-or-nothing thinking with exercise. However, there are several limitations, including the lack of racial, ethnic, and gender diversity in the sample. Most participants were white, with only a few men across the student and community focus groups. In addition, as an exploratory study we did not expect to discover any differences between life stages. However, as noted in the results, there were some nuanced differences between participants in the student compared to community focus groups, particularly with respect to college participants discussing less negative affect with exercising and less of an explicit desire to avoid it than participants in the community groups. We chose to build our initial framework (Fig. 1) based on the data from the community participants because they are more representative of the greater population than college students. Despite this difference, it was important to include the data from students because doing so creates a richer dataset for this initial qualitative study on exercise-related all-or-nothing thinking and can inform studies that are explicitly designed to discern potential differences related to all-or-nothing thinking with exercise across distinct populations, contexts, and life stages.

In addition, we examined all-or-nothing thinking among individuals who resonated with our recruitment messages, seeking participants who had tried to exercise but were unable to stick with it, because this group represents a significant proportion of the population [4]. Thus, these findings may not be relevant to individuals with no intentions to exercise or to individuals who successfully maintain an exercise routine. While we did not formally ascertain the number of times participants had attempted but failed to regularly exercise, when this topic arose in focus groups many participants affirmed that they had tried numerous times to start exercising but had been unable to stick with it. Despite these limitations, this research offers new insights into what may be a fundamental-but-overlooked barrier to regular exercise participation that has cognitive, motivational, and self-regulatory implications. It serves as a useful starting point for next-stage research and hypothesis testing about all-or-nothing thinking with exercise, a topic that has received little previous attention in the literature to date [26].

## Conclusion

The value of every choice is determined by its perceived value compared to the other alternative choices [15, 16, 58, 67]. Exercise-related all-or-nothing thinking may reflect a learned, socially prescribed rule and create a desirable escape route that guides unsuccessful exercise intenders to frequently choose to do *nothing* when their exercise intentions and plans face conflicts. Moreover, our analysis suggested that a symbiotic set of less obvious mental dynamics converged into a greater mindset that devalued exercising and supported all-or-nothing thinking among individuals who have tried but failed to regularly exercise. The exercise-related all-or-nothing mindset appeared to consist of an active desire to find an excuse to avoid negative effortful experiences and “should"-based exercise, a tendency for participants to sacrifice their rigid idealized exercise plans when facing other priorities they perceived as more important, and a lack of conscious awareness about the reasons behind their current lack of regular exercise. This study is an important first step toward better understanding and addressing exercise-related all-or-nothing thinking within public health, medical, and community-based interventions and initiatives.

## Supplementary Information

Supplementary material 1.

## Data Availability

De-identified data from this study are not available in a public archive. De-identified data from this study will be made available on reasonable request (as allowable according to institutional IRB standards) by emailing the corresponding author.
