# Supplementary material for: The secret life of all-or-nothing thinking with exercise: new insights into an overlooked barrier
Source: BMC Public Health. 2025 Dec 19;26:298. doi: 10.1186/s12889-025-25780-9 (PMC12831378; doi:10.1186/s12889-025-25780-9)
Supplement: Supplementary file 1 — Supplementary material 1. [file 12889_2025_25780_MOESM1_ESM.pdf]

Thinking about Our Experiences with Exercising and Being Physically Active Focus Group Protocol

Welcome, Get Food, and Getting Settled (10 mins)

HAVE RESPONDENTS READ/FILL OUT CONSENT WHEN WAITING TO BEGIN

6:10

Thank you for agreeing to participate in our discussion tonight about exercise and physical activity.

In front of you are two copies of an informed consent document. One is for you to sign and give back to us and one is for you to keep. Please read through and ask us any questions that you might have. I'd like to point out a few things:

- This is a research project, and your participation is voluntary.
- You may choose not to answer any question and you can choose to leave at any time during the session.
- The focus group questions and discussion will last about 60 to 90 minutes.
- After the discussion we'll ask you to complete a short questionnaire, and then we'll give you some resources and information about physical activity as well as compensation.
- The discussion will be audio-recorded and transcribed.
- Findings from the focus group will include the ideas that you all share with us today, but never your names.
- Please also keep information shared in the discussions today confidential, but note that we cannot guarantee your confidentiality because of the nature of a focus group.

We will provide you $50.00 pre-paid Visa gift card or course credit before you leave today as a thank you for your participation.

GATHER CONSENT FORMS **6:15**

Start of Focus Groups

Before we get started with our discussion, we would appreciate if you can silence the ringer on your cell phone; if you need to take a call, please step out and return as soon as you are done. We won't take a formal break,

but please feel free to step out if you need to, but don't all leave at once! If you need a restroom, they are located ___________.

I'm going to start the audio recording now. [TURN ON RECORDER]

I will call on you by your first name when appropriate. However, you don't have to wait for me to call on you. Feel free to speak naturally and interact with and respond directly to the other focus group participants. If we are starting to get short on time, we might have to interrupt the conversation to move to the next question.

As much as you can, please use each other's first names listed on the name cards so that we know who you're speaking to on the tape. Also, please try to speak one at a time so that can hear what everyone has to say.

It's important to know that there are no right or wrong answers to any of the questions. We want to hear about the different experiences and perspectives that people have had with exercising. Also, we might ask some similar questions in different ways throughout the discussion.

I am the Timekeeper. Because we have to make sure we get through all of the questions in time, I might have to interrupt even a great discussion for timing reasons. But, we hope to hear from everyone, even those of you who might be quiet!

Does anyone have any questions before we begin? [ANSWER ALL QUESTIONS]

6:17

Introductions *[5 minutes]*

Let's start off by going around the room: please state just your first name and your pronouns if you'd like, and tell us your favorite TV show or favorite show to stream. (*Tell people they can say "pass" if they don't want to share.)*

Now let's move to talk about exercising.

6:25

GENERAL REACTIONS ABOUT EXERCISING *[10 minutes]*

When you think about exercising or being physically active, what is the first word that pops into your mind? I'll go around the room and ask everyone to respond if you're willing. *[ask everyone}*

In general, what's your primary reason for wanting to exercise and why? *[ask everyone}*

What would a successful exercise routine look like for you?

Be strict on time here **6:35**

EXERCISE CYCLES *[30 minutes] – 8 questions*

We find that many people start and stop exercising and start and stop lots of different times. Can you please raise your hand if that sounds like your experience? [*state how many people raise their hands—state names of those in minority*]. Can you tell us about the ways in which this cyclical pattern of starting and stopping reflects – or doesn't reflect- your personal experience exercising?

Probe: If you didn't raise your hand, can you tell us about your experience?

Let's think about the process of starting to exercise. Please take a minute to remember a time when you've started to try to exercise. (PAUSE). At the times you've tried to start exercising again, what do you do for exercise? *[Pause before going to the prompts below.]*

Prompt if needed: For example, what activity or activities do you typically choose to do when you try to start exercising?

How long do you typically plan to exercise for?

How how many times per week do you typically aim to exercise for?

Specifically at the times you've started exercising, what is your *primary* reason for starting and why?

Does exercise feel more like something that is important to you personally or more like something you "should" do, and why? *[try to hear from a lot of people]*

We just talked about starting to exercise, so now let's shift gears to think about the other part of this cycle, which is when exercise gets hard to stick with. Please take a minute to remember a time when exercise became challenging and you stopped exercising. (PAUSE). At these moments when exercise becomes challenging, what types of things make it hard to stick with it? (*ask everyone*, encourage to explain as much as possible.)

We're talking about starting and stopping exercise. Since exercise can be challenging to stick with, why do you think you keep trying to exercise?

7:05

PLANS FOR EXERCISING *[10 minutes]—3 questions*

Now let's talk more about *the process of* starting to exercise. Once you've decided to start exercising, how do you make it happen?

Probe: For example, do you plan it into your schedule or decide to do it without making any specific plans?

How often do you plan a specific time and place to exercise, and why or why not?

When you've made a plan to exercise, do you typically follow through with it and why or why not? (can skip if necessary)

7:15

DISRUPTORS OF PLANS *[30 minutes]—6 questions*

Let's imagine that you've made a plan to exercise on a specific day. It's time for you to start exercising or to leave to get to the place where you planned to exercise. Take a moment to think about this situation. [Pause]. At that moment, what types of things tend to get in the way of actually exercising?

In general, when something unanticipated happens that prevents you from doing *exactly* what you planned to do for exercise, what do you tend to do?

At those times when something gets in the way, how often do you change the activity or the duration of what you were planning to do? *(ask everyone)*

If not, why not?

At those times when *you don't feel motivated to exercise*, how often do you change the activity or the duration of what you were planning to do?

Now I'm going to give you a specific example: Let's say that you're about to start your planned exercise or about to leave to go do it. Imagine you get an unexpected text that requires you to do something right then that takes 20 minutes. What do you typically do about that planned exercise session?

Probe if needed: Would you still decide to exercise then? Why? Why not? (go around room if previous question didn't sufficiently get respones.)

Sometimes, people say they have "back up plans" in case something gets in the way of their original exercise plan. Please raise your hand if you tend to have back up plans *[state names for record].* Can you tell me about why you do or do not have back up plans?

If yes: What do your back up plans tend to look like?

7:45

If time:

Do you think you are too ambitious or not ambitious enough when it comes to exercising—how so?

We've been talking only about back up plans for exercising and whether you have them. Now we want to take a big step back and ask if there are areas in your life—other than exercise—where you do have back-up plans if something gets in the way of what you were planning to do?

> Probe/example: if you need to study for a test, or were planning to hang out with a friend, cooking

When you think about making back-up plans, is exercise similar or to or different from these other areas of your life?

FINAL PROBE *[? minutes] **not planning to ask*

Those are all the questions we have for you. Do you think we missed asking anything that you think could be important for us to know about before we end?

QUESTIONNARE – DEMOGRAPHICS + QUESTIONS OF INTEREST *[15 minutes]*

We have a questionnare for you to fill out that should take about 10 minutes.

Please read the instructions for the different questions and just raise your hand to get our attention if you have any questions.

When you finish the survey, you can come over to the table and we'll have two things to give you.

First, we'll give you a document with resources about physical activity.

We'll also give you the $50 pre-paid Visa gift card as a thank you for your participation and ask you to sign a document acknowledging that you received the compensation.

After that you are free to go.

Thank you for your thoughts and your time today—this has been extremely helpful and we really appreciate it.

****Have them sign receipt for gift card.**

8:00

THANK YOU

Do you have any other questions?

Debriefing if do it:

People ask us if there's a best way or right way to exercise, and the answer is there is no right way. the best way is what feels good to you and what fits into your life. it's all about you taking ownership.
